# Assessing the utility of experimentally derived upper thermal limits to predict vulnerability of marine macrophytes to future ocean warming

**DOI:** 10.1038/s41598-025-23074-z

**Published:** 2025-11-10

**Authors:** Kathryn E. Smith, Nathan G. King, Margot Aubin, Tayla Leathers, Dan A. Smale

**Affiliations:** The Marine Biological Association of the United Kingdom, The Laboratory, Citadel Hill, Plymouth, PL1 2 PB UK

**Keywords:** Temperature, Climate change, Kelp, Fucoid, Seagrass, Thermal thresholds, Climate sciences, Ecology, Ecology, Environmental sciences, Ocean sciences

## Abstract

Marine foundation species such as large macrophytes (i.e. seagrasses, canopy-forming seaweeds) serve critical ecological functions, yet are increasingly impacted by anthropogenic climate change. Temperature extremes pose significant challenges to these species, and identification of thermal thresholds offer valuable insights into how populations and species may respond to current and future climatologies. The objectives of this study were to compile experimentally-derived upper thermal limits (T_limit_) of habitat-forming marine macrophytes into a centralized database, enabling further research. We conducted a Web of Science and Google Scholar search in June—August 2024 with three review participants to avoid bias. Any studies where a T_limit_ value could be identified for kelps, seagrasses or fucoids globally met the inclusion criteria for the study. Studies where T_limit_ could not be determined were excluded. After full text extraction, our data was analysed by frequency with regards to study region, target species, methodological approaches and other descriptors. The most extensively studied species were further examined to explore intraspecific associations with these descriptors. A comprehensive database of these values was collated and interrogated for marine macrophytes globally, incorporating 365 T_limit_ estimates extracted from 63 studies and representing 75 species. For seaweeds (kelps and fucoids), T_limit_ values ranged from 15 to 36 °C, whereas estimates for seagrasses ranged from 25 to 48 °C. T_limit_ values increased with decreasing latitude, both within and between species, and were influenced by life history stage, experimental duration and response variable chosen to determine thresholds. The existing database is characterised by several biases, including geographical regions and target species. Variation and uncertainty in reported T_limit_ values hinder our ability to predict suitable thermal habitats for marine macrophytes. Improved understanding of upper thermal thresholds is needed to improve predictions of current and future impacts of ocean warming, which will inform approaches to management and conservation of wider coastal communities and ecosystems.

**Supplementary Information:**

The online version contains supplementary material available at 10.1038/s41598-025-23074-z.

## Introduction

Temperature is a key driver of all processes operating across biological scales, from genes to ecosystems, as species have evolved to occupy specific thermal niches within which they can function and persist^1,2^. However, global ocean temperatures have increased rapidly as a consequence of anthropogenic climate change, shifting the geographical location of thermally-suitable habitat and driving species range shifts^3–5^. These shifts are in turn causing changes in the structure and functioning of communities and ecosystems and threatening the provisioning of ecosystem services to societies^3,6,7^. In response to gradual decadal-scale warming, range shifts often manifest through altered fitness over multiple generations, leading to population-level changes^5^. However, when extreme warming events occur (i.e. marine heatwaves, see^8^), temperatures can quickly exceed critical thermal thresholds, resulting in rapid, profound effects such as widespread mortality, population crashes and local-to-regional extirpations^9–13^. Marine heatwaves have increased in frequency, intensity and duration over recent decades when considered against a fixed historical baseline^14,15^, and have been particularly prevalent globally over the past few years^16^. As such, they are an increasingly prominent stressor impacting marine environments and the organisms within them, in particular for slowly adapting species which are likely to evolve at slower rates than the rapidly changing climate^15^. If foundation species, such as corals, seagrasses and seaweeds are affected by either background warming or marine heatwaves, the knock-on consequences for the wider community and ecosystem can be dramatic due to their fundamental roles in maintaining ecological processes^17,18^. Given both gradual ocean warming and marine heatwaves are projected to intensify further over the coming decades^19,20^, there is an urgent need to determine when and where thermal thresholds will be exceeded, to predict population and community-level impacts and develop pre-emptive management and conservation strategies. In particular this is relevant given recent evidence of negative impacts of marine heatwaves on foundation species increasing over time in line with marine heatwave intensification^21^.

The most widespread tools for predicting climate-driven species range shifts, such as species distribution models, generally work by establishing statistical relationships between present day species observations and underlying environmental variables, such as average sea surface temperature. Given these are based on static long-term means rather than climatological variability^22,23^, they offer limited information on species responses to extreme temperatures and the rapid and acute fluctuations of species distributions, particularly at range edges where species are already living close to their thermal limits. To address this, there has been increasing focus on developing mechanistic models that incorporate underlying organismal physiology^24–26^, although this is often limited by the substantial experimental effort and technical capabilities required to obtain such information. The study of critical thermal limits has a rich and extensive history in marine ecophysiology and, as such, there may be opportunities to overcome these challenges using evidence from the existing literature. However, there is no standardised approach for measuring thermal limits and technical differences between studies have the potential to obscure patterns and limit their real-world utility. Consolidation and exploration of experimentally-derived data on species upper thermal limits is a critical step towards building predictive tools for understanding the impacts of ocean extremes, while also highlighting biases and limitations that should be considered in developing best practise for future research.

Marine macrophytes (seaweeds and seagrasses) are coastal foundation species that play a fundamental role in maintaining core ecological processes and exerting disproportionate influences on associated communities and the wider ecosystem^17,18^. They are widely distributed along the world’s coastlines, where they enhance biodiversity^27–29^, alter local environmental conditions^30^, provide nursery grounds for a range of commercial species^31,32^ and contribute to primary productivity and inshore carbon cycles^33,34^. Their distributions are strongly constrained by temperature meaning they are particularly sensitive to ongoing and anticipated warming trends^21,35,36^. Episodic marine heatwaves can accelerate and amplify these responses, leading to rapid shifts in macrophyte abundance and distribution, generally towards warmer-affinity species^10,21,37–42^. As such, consolidation of marine macrophyte thermal limits will be an asset for those responsible for managing these systems, with the caveat that ocean warming does not impact species in isolation but rather in conjunction with other concurrent stressors that interact to drive ecological changes^43^.

The objectives of this study were to collate experimentally-derived estimates of the upper thermal limits of habitat-forming marine macrophytes (i.e. kelps, fucoids, seagrasses) to provide a centralised database to facilitate further research, and to interrogate this database to explore inter and intraspecific variation in upper thermal limits, and to highlight biases, limitations and priorities within the research area. Such systematic reviews represent an effective approach to synthesising data and providing summaries of findings, making them particularly useful for informing research priorities, conservation planning and decision-making. We focussed primarily on laboratory-based physiology experiments, excluding those based on field observations from natural settings (e.g. from a mass die-off in response to a marine heatwave), as critical thermal limits could not be precisely inferred. Thermal threshold values that had been estimated from growth models were also included (e.g.^44^).

## Material and methods

### Literature search and data compilation

A literature search was conducted between 3^rd^ June and 30^th^ August 2024 using Web of Science (www.webofscience.com) and Google Scholar (www.scholar.google.com) with the keyword combinations (*kelp OR macroalgae OR seaweed OR seagrass OR fucoid) AND (temperature OR warming) AND (“thermal limit” OR “thermal threshold” OR “critical temperature” OR “thermal niche” OR “CT*_*max*_*”).* K.E. Smith, M. Aubin and T. Leathers performed the search and screened results independently to reduce bias. Search terms were adequately broad to return papers across the macrophyte groups and time period and further searches of key authors, species and collections indicated that initial searches were near-exhaustive. These manuscripts were then vetted, as well as any relevant papers identified in reference lists, and all identifiable published upper thermal limit (T_limit_) for all available species of kelp, fucoid and seagrass were compiled. For the purpose of this study, T_limit_ was defined as the temperature prior to which response variable measured zero (for growth, photosynthesis or reproduction) or when a maximum of 10% survival was observed. Values were extracted from figures, tables and text, and where different definitions of T_limit_ were used within a study, we determined the most appropriate value following the above definition. Although ecologically-relevant sub-lethal impacts may occur at lower temperatures, as organism recovery and persistence is generally possible only T_limit_ values were extracted and analysed in the current study, to avoid overestimating the vulnerability of species to warming. If reliable T_limit_ values could not be determined, the study was rejected from the analyses. In addition to ‘true’ kelps (i.e. species belonging to the order *Laminariales*) some kelp-like species (e.g. the Tilopteridales *Saccorhiza polyschides* and *Saccorhiza dermatodea*) were also included in the search as they serve similar functions as foundation organisms in coastal ecosystems. Each record added to the database was screened by three reviewers.

### Extracted metadata

For each study, the reported values for upper thermal limit (T_limit_, often reported as critical thermal maxima or CT_max_) were extracted as well as key contextual information, including: target species, location of the population studied, year of study, experimental conditions (e.g. acclimation period, heating rate, temperature treatments and duration, time of year) and biological factors (response variable measured, life history stage). Experimental conditions were defined as follows: acclimation period refers to the period of time plants were maintained in aquaria under static conditions prior to experiments beginning, heating rate was considered ‘instant’, ‘hours’ or ‘days’ depending on the time frame over which the experimental temperature was reached, and time of year was recorded as ‘spring’ (Northern Hemisphere [NH]: Mar-May, Southern Hemisphere [SH]: Sep-Nov), ‘summer’ (NH: Jun-Aug, SH: Dec-Feb), ‘autumn’ (NH: Sep-Nov, SH: Mar-May) or ‘winter’ (NH:Dec-Feb, SH: Jun-Aug). Biological factors of interest were the response variable being measured and was defined as ‘photosynthesis’, ‘growth’, ‘fecundity’, or ‘survival’, and life history stage was recorded as either adult (for sporophytes or shoots/plants) or juvenile (for gametophytes or seedlings).

### Trends and analyses

Trends and biases in the database were first examined with descriptive statistics, to determine the proportion of studies and/or T_limit_ values relating to different study regions, target species, methodological approaches and other descriptors. Linear regression were then used to explore relationships between upper thermal limits and latitude across all species. For species with sufficient independent estimates of T_limit_ (n ≥ 10) linear regressions were used to explore relationships between upper thermal limits and latitude, and one-way ANOVA to examine differences between key methodological (i.e. experimental duration) and biological (i.e. life history stage and type of response) factors. For multiple independent tests, the Bonferroni correction was applied to maintain an overall α of 0.05. The P-values for the individual tests are presented with their corresponding adjusted rates, α_adj_. Data were checked for homoscedasticity and normality prior to analysis and were transformed where necessary.

## Results

### Metadata

**Fig. 1:**
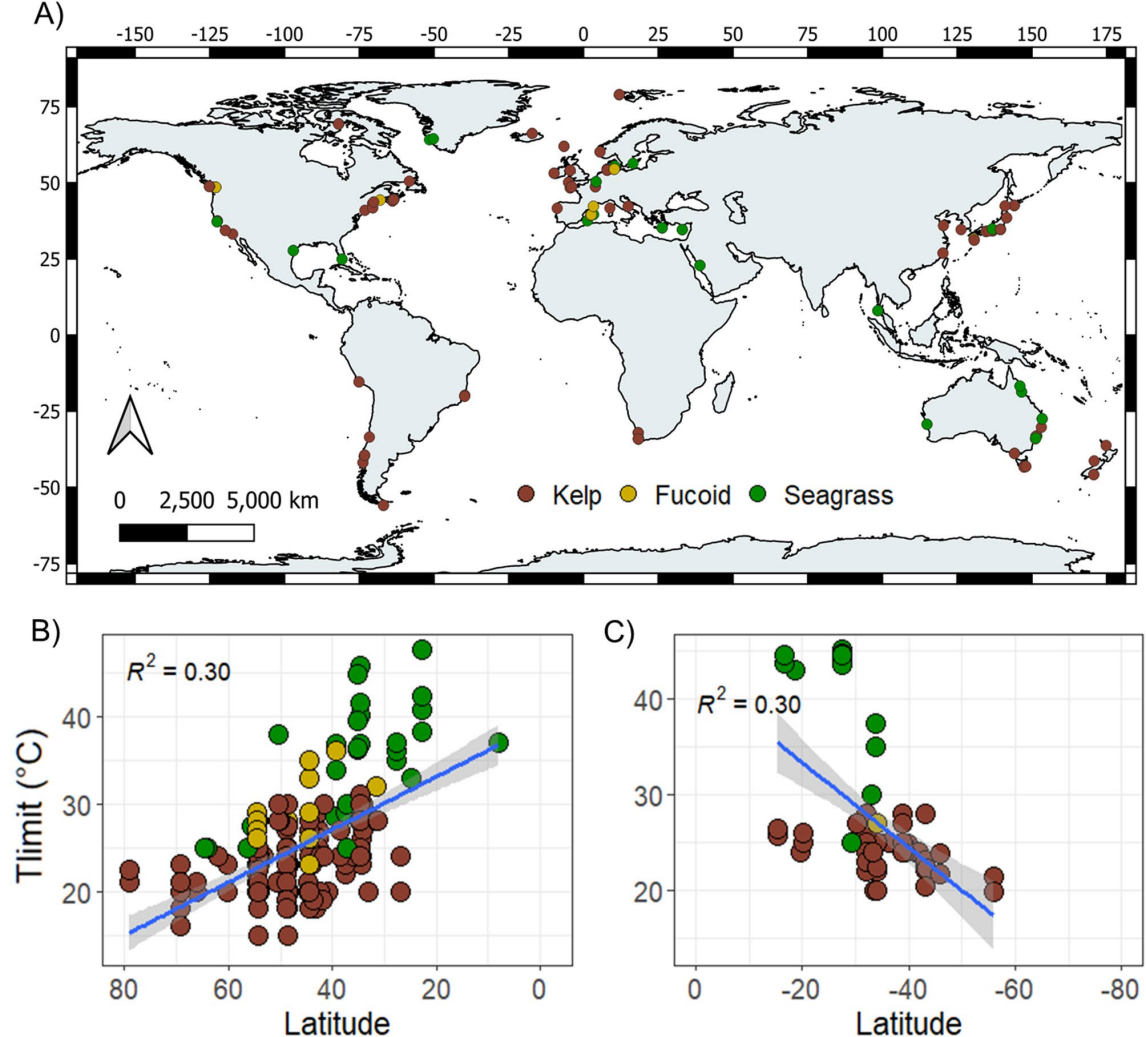
(**A**) Locations of marine macrophyte thermal tolerance studies. B & C) Linear regression indicating relationships between T_limit_ and latitude across all macrophytes in the northern (**B**) and southern (**C**) hemispheres. For both linear regressions, *p* ≤ 0.001.

**Fig. 2:**
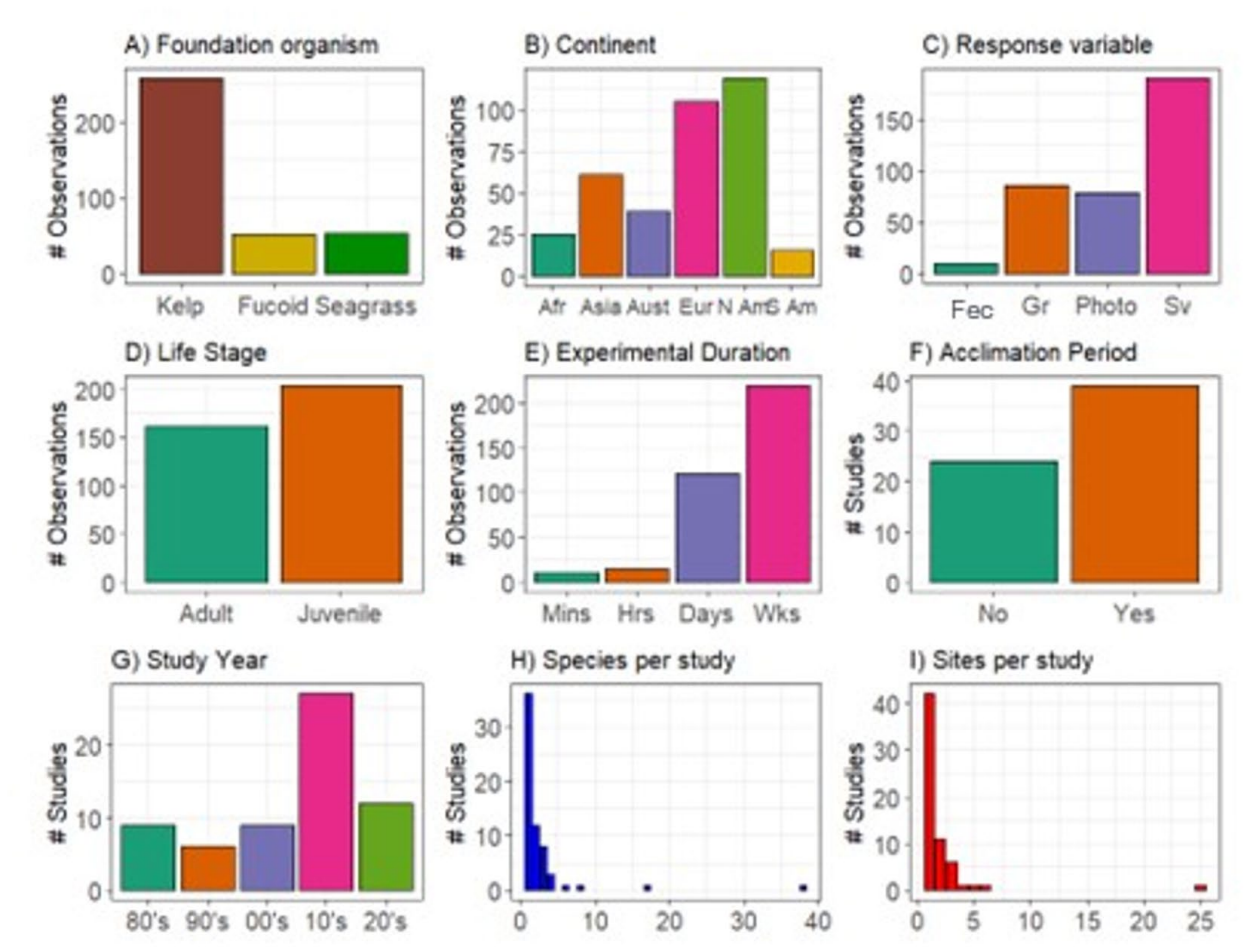
Overview of trends and biases in the marine macrophyte thermal tolerance literature. (**A**) number of target species for each type of foundation organism, (**B**) location of target species, by continent (Afr: Africa; Asia: Asia; Aust: Australia; Eur: Europe; N Am: North America; S Am: South and Central America), (**C**) response variable examined for each species (Fec: fecundity; Gr: growth; Photo: photosynthesis; Sv: survival), and (**D**) life stage examined for each species. Also shown are (**E**) the experimental duration of each study, (**F**) the numbers of studies that specifically included an acclimation period, (**G**) the year of study, and (**H**) the number of target species and (**I**) different sites/populations examined within each study.

A total of 63 papers published between 1982 and 2024 were identified that met the criteria for inclusion in this study (Supplementary Table 1, supplementary Fig. 1). The studies had a broad global distribution (Fig. 1a) and described 75 species including 48 species of kelp, 11 species of fucoid and 16 species of seagrass (Supplementary Table 1). In total, 365 estimates of upper thermal limit were extracted (71% derived from kelp species), which showed cleared trends in the literature (Fig. 2). Most studies (61.9%) were published after 2010 and typically included one species (57.1%) and one location (66.7%). Of the 365 estimates of T_limit_, most focussed on species from the Northern Hemisphere (78.3%), in particular North America (32.3%) and Europe (29.1%). The majority of estimates were for kelp (70.3%), for juvenile life stages (55.2%) and included survival as the main response variable (52.4%). Most studies (64.9%) employed an acclimation period and the experiments themselves most commonly ran for ‘weeks’ (59.5%) as opposed to shorter durations (Fig. 2). Across all species combined, linear regression indicated that upper thermal limit increased significantly with decreasing latitude in both hemispheres (*p* < 0.001, R^2^ = 0.30 for both; Fig. 1b,c).

### Interspecific variability

**Fig. 3:**
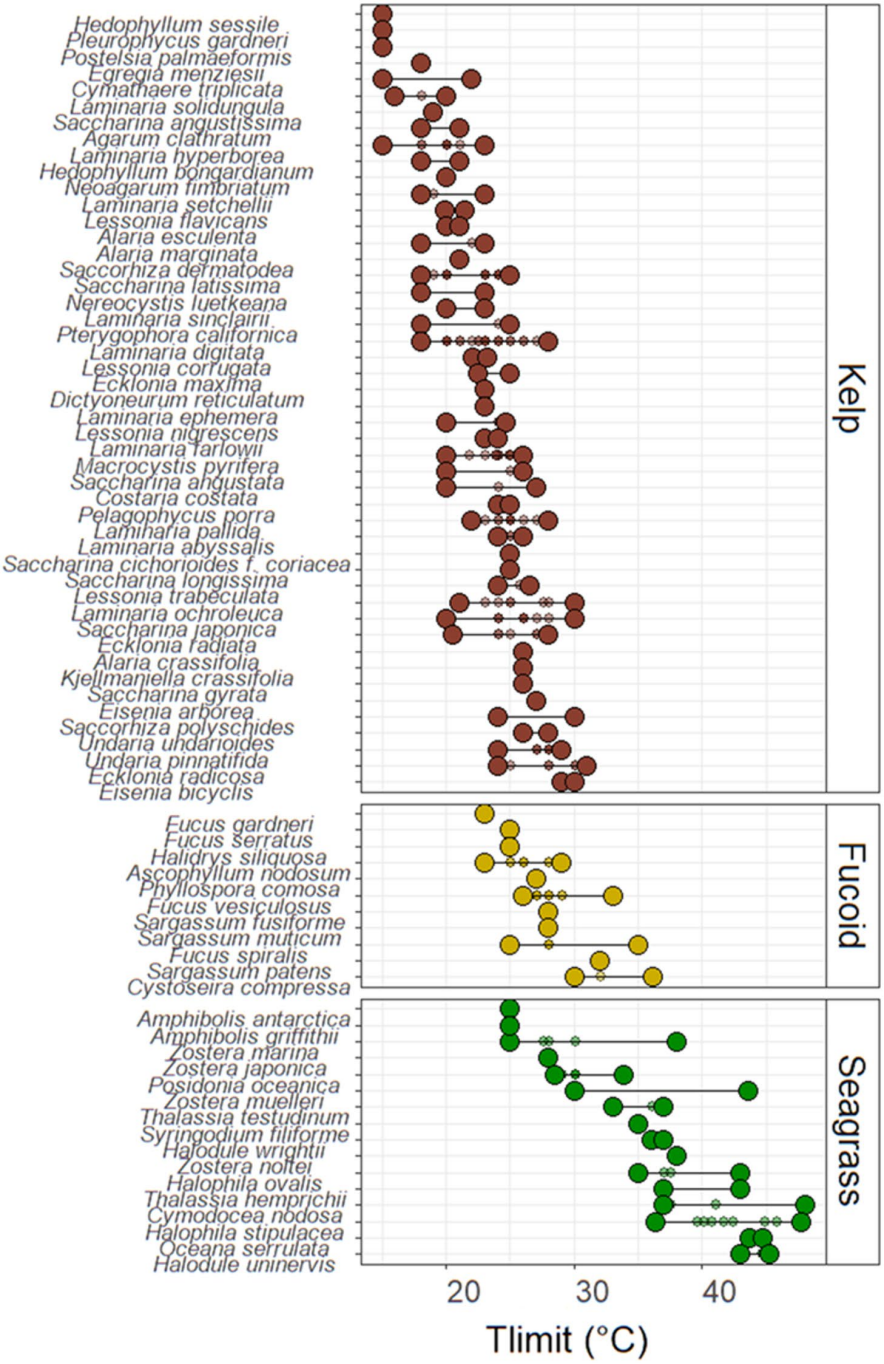
Individual T_limit_ values obtained from the literature for kelps, fucoids and seagrass. Bar indicates range of values. Large circles indicate minimum and maximum values. Small circles indicate other datapoints.

Across all of the macrophytes, T_limit_ values were generally lowest for the kelps, followed by the fucoids and then the seagrasses, although pronounced variability both within and among species was recorded (Fig. 3). For the kelps, the lowest upper thermal threshold values were observed for *Hedophyllum sessile, Pleurophycus gardneri* and *Postelsia palmaeformis*, which all had T_limit_ estimates of ~ 15 °C. The highest upper thermal threshold values were observed for species including *Eisenia bicyclis* (T_limit_ 29–30 °C)*, Ecklonia radicosa* (T_limit_ 24–31 °C), *Saccorhiza polyschides* (T_limit_ 24–30 °C) and *Undaria pinnatifida* (T_limit_ 24–29 °C). As such, inter-specific estimates of T_limit_ for kelps ranged by more than 15 °C. Interspecific variability in T_limit_ for fucoids was similar, spanning more than 13 °C, although estimates were generally higher than for kelps. The lowest upper thermal threshold values were identified for species including *Fucus gardneri*, *Fucus serratus* and *Halidrys siliquosa* (T_limits_ of 23 °C, 25 °C and 25 °C, respectively). The highest upper thermal threshold values were seen in species including *Cystoseria compressa* (T_limit_ 30–36 °C)*, Sargassum patens* (T_limit_ 32 °C) and *Fucus spiralis* (T_limit_ 25–35 °C)*.* For seagrass, upper thermal threshold values were lowest for *Amphibolis antarctica* (T_limit_ 25 °C)*,* and *Amphibolis griffithii* (T_limit_ 25 °C)*,* and highest for *Halodule uninervis* (T_limit_ 43–45 °C)*, Cymodocea nodosa* (T_limit_ 37–48 °C) and *Halophila stipulacea* (T_limit_ 36–48 °C). Overall, estimates of T_limit_ varied by 23 °C across seagrass species. Within single species where multiple data points were available (Fig. 3), variation in T_limit_ ranged from less than 1 ˚C (observed for *Dictyoneurum reticulatum*, *Saccharina cichorioides, Saccharina longissima*, *Alaria crassifolia*, *Kjellmaniella crassifolia*, *Saccharina gyrata*, *Eisenia arborea* and *Fucus serratus*) to more than 13 ˚C (*Zostera muelleri*).

### Intraspecific variability

**Fig. 4:**
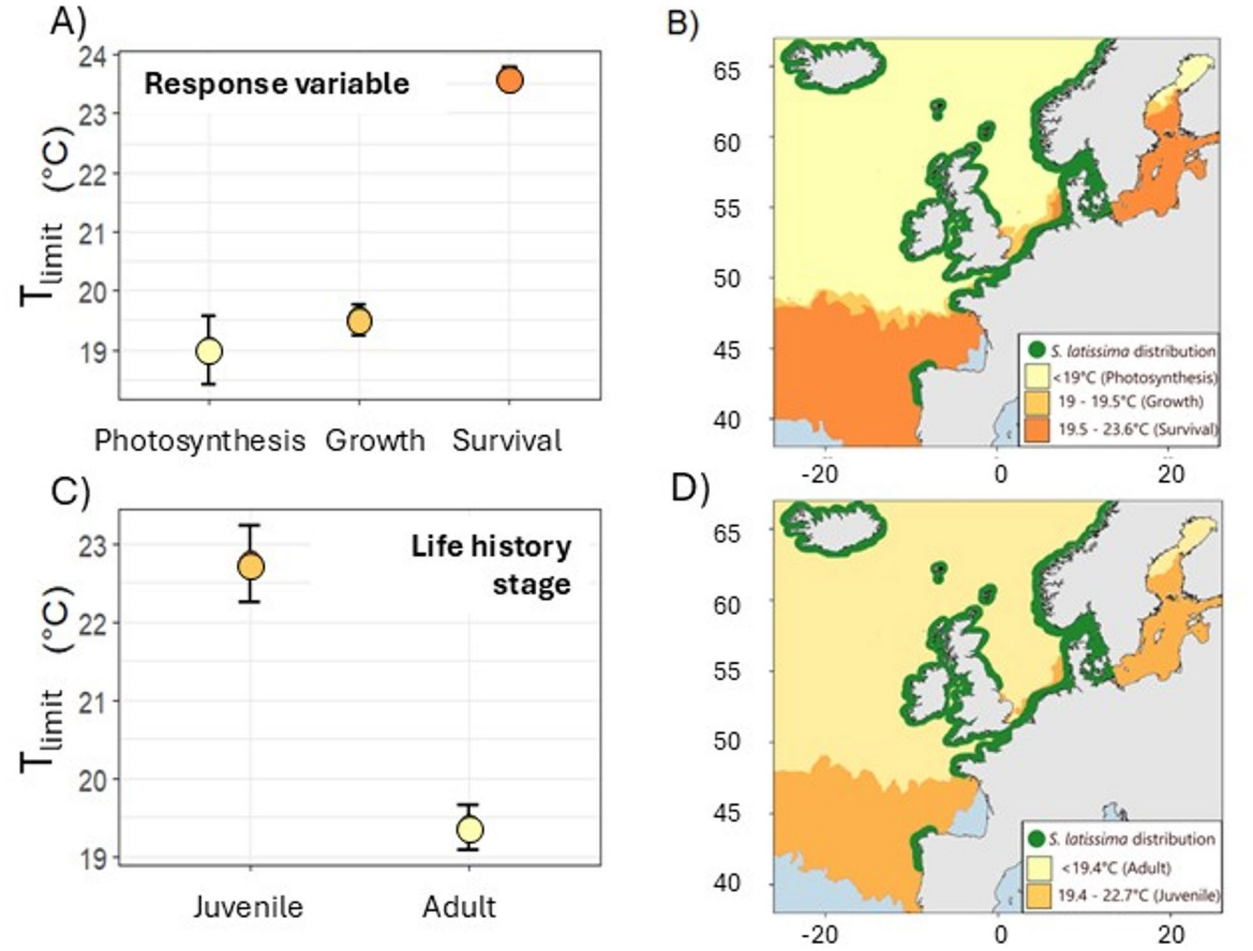
Differences in identified T_limit_ values for *S. latissima* for (**A**) response variable measured, and (**C**) life history stage. (**B**) and (**D**) Map of the northeast Atlantic showing the current approximate distribution of *S. latissima* (based on occurrence records from GBIF) and areas where maximum SST falls within the T_limit_ values identified in (**A**) and (**B**). Maximum SST values were obtained from the NOAA OISST dataset, averaged over a ten-year period (2012–2022).

For eight species it was possible to extract and collate ≥ 10 estimates of T_limit_; these were selected for further exploration of intraspecific variability in upper thermal thresholds. Of these, variability in T_limit_ between experimental duration was formally examined for eight species, between response variables for seven species, and between life history stage for the five species for which enough information was available. Experimental duration had a significant effect on five out of the eight species examined, with longer duration experiments typically resulting in lower upper thermal thresholds (Table 1). For four out of the seven species examined, the response variable chosen to determine the upper thermal threshold had a significant effect on T_limit_ values (Table 1). In most cases, survival and photosynthesis yielded greater T_limit_ values than growth or fecundity. For the five species tested for the effects of life history stage on T_limit_ estimates, one (*Saccharina latissima*) returned a significant result (Table 1). Here, the upper thermal limit estimates were higher for juveniles (that is, gametophytes) compared with adults. The influence of different experimental durations, response variables and life history stages on estimates of T_limit_ was particularly evident for the kelp *Saccharina latissima*. Average T_limit_ varied by 4.6 ˚C across different response variables, by 2.8 ˚C when obtained from experiments lasting weeks rather than days, and was 3.3 ˚C higher for juveniles compared with adults (Fig. 4). When transposed onto a map of maximum sea surface temperatures for the northeast Atlantic, the higher T_limit_ values would suggest a considerably greater area of thermally-suitable habitat and are more representative of the current approximate distribution of *S. latissima* than the lower T_limit_ estimates (Fig. 4).

**Table 1:** Results of one-way ANOVAs comparing estimates upper thermal thresholds between experimental duration (h = hours, d = days or w = weeks), response variables (f = fecundity, g = growth, p = photosynthesis, s = survival) and life-history stage (a = adult, j = juvenile) within a species. The *P*-values for the individual tests are presented with their corresponding adjusted rates, α_adj_. Significant *p*-values are in bold.

Species	df	F	p	α_adj_	
**Measured response variable**					
*Ascophyllum nodosum*	2	0.929	0.426	0.017	–
*Fucus vesiculosus*	2	5.348	**0.015**	0.017	s = p > g
*Laminaria digitata*	2	4.391	0.019	0.017	–
*Laminaria hyperborea*	1	7.492	**0.023**	0.025	s > p
*Macrocystis pyrifera*	1	2.996	0.107	0.025	–
*Saccharina japonica*	2	14.03	**0.002**	0.017	s = p > g
*Saccharina latissima*	2	86.62	** < 0.001**	0.017	s > p = g
**Duration**					
*Ascophyllum nodosum*	1	1.523	0.243	0.017	–
*Fucus vesiculosus*	1	31.45	** < 0.001**	0.017	d > w
*Laminaria digitata*	2	2.988	0.062	0.017	–
*Laminaria hyperborea*	1	7.44	**0.021**	0.025	w > d
*Laminaria pallida*	1	11.95	**0.003**	0.05	d > w
*Macrocystis pyrifera*	1	3.329	0.090	0.025	–
*Saccharina japonica*	2	11.25	**0.004**	0.017	h > d = w
*Saccharina latissima*	1	13.52	**0.001**	0.017	w > d
**Life history stage**					
*Ascophyllum nodosum*	1	1.523	0.243	0.017	–
*Fucus vesiculosus*	1	5.503	0.030	0.017	–
*Laminaria digitata*	1	3.633	0.064	0.017	–
*Saccharina japonica*	1	0.150	0.708	0.017	–
*Saccharina latissima*	1	29.08	** < 0.001**	0.017	j > a

For one species, *Laminaria digitata*, it was possible to explore variability in T_limit_ across latitude as a sufficient number of estimates derived from the same response variable and life stage were available. Here, T_limit_ increased significantly with decreasing latitude (R^2^ = 0.34; *P* < 0.05).

## Discussion

The increase in frequency and severity of climatic extremes as a consequence of anthropogenic climate change represents a pervasive stressor acting on contemporary ecosystems^45,46^. In coastal ecosystems, extreme temperatures experienced during recent marine heatwaves have had far-reaching effects on species and communities, ecological functions and processes, and the provisioning of ecosystem services for society^17,37,39,47^. As such, there is a pressing need to combine natural observations with experimental research to better understand the capacity of key organisms, such as foundation species, to withstand anomalously high temperatures. We collated existing information on experimentally-derived upper thermal limits of globally-distributed marine macrophytes to discern trends, biases and knowledge gaps. Understanding upper thermal limits is essential for predicting current and future warm limits of species distributions^48,49^ and identifying temperature thresholds above which population losses may occur in the near-term^9,50^. Consequently, measures of T_limit_ are increasingly being used to estimate changes in species distributions and to guide conservation efforts and management practices^51,52^.

Our analysis of compiled T_limit_ estimates revealed considerable variability between both species and types of foundation organism, with values ranging from 15 to 48 ˚C. This wide range is indicative of the extreme breadth of temperature tolerance of marine macrophytes, which are widely distributed from polar to tropical regions where they play key roles as foundation species^53,54^. The evolution of the brown algae lineage began ~ 250 Ma, although fucoids and kelps radiated more recently (145–66 Ma), quickly diversifying to ~ 500 and ~ 110 extant species, respectively^55,56^. In contrast, seagrasses evolved later (~ 100 Ma), and exhibit lower diversity, with ~ 60 extant species of marine angiosperms^57,58^. Even so, they have evolved within six distinct bioregions, spanning the cool temperate North Atlantic and Southern Oceans through to the tropical Indo-Pacific, to now occupy highly divergent thermal habitats^58^. As such, the marked interspecific variation in T_limit_ recorded across all marine macrophytes is likely reflective of their diverse evolutionary histories and biogeographic distributions^53,58,59^, and explains in part the correlations observed between T_limit_ and latitude in both hemispheres.

Although highly variable both within and among species, there was a general pattern of kelps exhibiting lower T_limit_ values than fucoids, which in turn were lower compared with seagrasses. This trend reflects their broad evolutionary history and biogeographic distributions, as kelp typically occupy polar or cold-temperate regions whereas most seagrass species are found in warm-temperate or tropical regions^58,59^. Fucoids exhibit comparatively high variability in thermal affinities, with several diverse genera (e.g. *Fucus*) being largely restricted to cooler regions and others (e.g. *Sargassum*) being primarily found in warmer waters^59,60^. Amongst other factors, habitat preference and local environmental temperatures are likely important determinants of T_limit_, as has been shown for both tropical^61^ and temperate^62^ marine macrofauna. For example, kelp species are restricted to subtidal or extreme low shore environments, where temperatures are relatively stable and few stressful thermal extremes are experienced^63^, whereas many fucoid and seagrass species extend into mid to high intertidal environments where they routinely experience high thermal stress and variability^64,65^.

For the marine macrophyte species that were relatively well studied, such as the kelps *Saccharina latissima* and *Laminaria digitata*, the fucoids *Ascophyllum nodosum* and *Fucus vesiculosus* and the seagrasses *Zostera marina* and *Halophila stipulacea*, considerable variability (often > 10 ˚C disparity) was observed in reported T_limit_ values for a given species. On further examination, we found that the choice of response variable significantly affected T_limit_ estimates in 57% of species examined, experimental duration in 63%, and the selected life stage in 20% of species. For the chosen variable, sublethal responses such as fecundity and growth exceeded tipping points at lower temperatures than photosynthesis and survival. Intuitively, different response variables are underpinned by distinct biochemical and physiological processes, which will be influenced by high temperatures in distinct ways. Across response variables, the underlying processes are not directly coupled and consequently stress responses to high temperatures may manifest differently^66,67^. For example, a sudden reduction in photosynthesis typically occurs once a thermal threshold is exceeded, due to factors including temperature sensitivity of enzymes for carbon fixation, photophosphorylation and the thermal stability of photosystem II^68,69^. In contrast, as growth is a more general process that integrates all positive and negative influences of temperature on the overall metabolism of an organism, changes in growth and tissue maintenance may occur at lower temperatures or across different timescales^68,70^. T_limit_ estimates derived from survival rates are intuitively higher as temperature-related mortality ensues once all other physiological processes and protective mechanisms fail. Variability across different responses to thermal stress for a given species exposed to the same temperatures has been shown for both seagrasses^71^ and seaweeds^66^ and will strongly influence estimates of T_limit_.

The significant effect of experimental duration on T_limit_ estimates can also be explained by underlying biological mechanisms. Shorter periods (i.e. hours or days compared to weeks) often resulted in higher upper thermal limits, as marine macrophytes can temporarily cope with high temperatures for short periods without ecological performance being impacted^64,65,72^. Over longer periods (i.e. days), phenotypic plasticity may facilitate acclimation to higher temperatures and protective mechanisms, like heat shock proteins, may be employed to maintain function^73,74^. However, such mechanisms are metabolically costly causing energy reserves to be rapidly depleted and, over even longer periods (i.e. weeks), thermal tolerance may decline^69^, as has been shown for both macrophytes^71,75^ and other marine ectotherms^76–78^. Clearly, the experimental duration, temperature ramping rate and inclusion (or lack) of acclimation and recovery phases will strongly influence estimates of T_limit_ for marine macrophytes.

Variability in T_limit_ across life history stages was also observed for one kelp species (*Saccharina latissima*), whereby gametophytes exhibited greater thermal tolerance than sporophytes. Kelp gametophytes have been shown to exhibit broad tolerance to environmental conditions, including temperature^79^, suggesting resilience to ocean warming and other environmental change factors^80,81^. Improved understanding of upper thermal limits across different life stages is needed to more accurately predict responses of macrophytes to climate change, as has been shown for other marine taxa^82,83^. Furthermore, for one species (the kelp *Laminaria digitata*) a significant relationship was found between decreasing latitude and increasing T_limit_, implying to some extent, that populations located towards the species warm range edge may be more tolerant of acute temperature stress, perhaps suggestive of ‘thermal divergence’ through phenotypic plasticity or local adaptation^84^. Intraspecific variability in thermal tolerance has been demonstrated experimentally for *L. digitata*^85,86^ and other kelps^87,88^ and may be a feature of marine macrophytes more generally^84^. Clearly, further research on population-level vulnerability to extreme temperatures is needed to predict the wider impacts of ocean warming.

Quantifying thermal tolerances of a wide range of organisms has become a priority in the field of climate change ecology, to inform predictions of responses to both gradual warming and extreme climatic events^62,89^. The compiled database of upper thermal limits for marine macrophytes provides a useful resource to: (i) identify knowledge gaps to plan and prioritise experimental research, (ii) generate testable hypotheses regarding ecophysiological processes and ecological trends; (iii) evaluate the usefulness of different methodological approaches in estimating T_limit_; (iv) inform mechanistic process-based population and species distribution models; and (v) assess the risk of macrophyte species and populations to current and predicted future ocean temperatures. However, this database highlighted several trends and limitations that may restrict the ecological relevance and usefulness of T_limit_ within the context of predicting climate change impacts on marine macrophytes. First, as with thermal tolerance research conducted on fishes^89^, the experimental approach taken for marine macrophytes will influence the relevance for real-world predictions, as factors such as acclimation temperature and duration, rate of thermal ramping, experimental duration and season will affect T_limit_ estimates and should therefore be as realistic as possible. Second, T_limit_ estimates are a measure of susceptibility to acute temperatures rather than thermal stress, and reflect short-term biochemical and physiological processes rather than longer term organismal and demographic responses to warming, which are important in determining climate change impacts on marine macrophytes^90,91^. Third, seaweeds and seagrasses represent highly diverse and widely distributed taxa, which complicates comparisons and generalisations both within and among species. Moving forward, a more systematic and comparable approach to determining upper thermal limits, using standardised protocols for example, would significantly advance wider understanding of their vulnerability to ocean warming.

More generally, coastal environments are highly complex and dynamic, and responses of organisms in their natural habitats to acute or chronic thermal stress will be mediated by many factors that are not captured within typical ‘simple’ thermal tolerance experiments. For example, extreme temperatures experienced during marine heatwaves often co-occur with changes in other variables, such as oxygen, nutrients or light levels, which are known to mediate responses to thermal stress^92–94^. Similarly, the climatic history of a particular location or region may influence upper thermal limits, as repeat exposures to high temperatures potentially increase thermal tolerance through ‘priming’^95,96^ or, conversely, erode resilience and reduce thermal thresholds^97,98^. Moreover, thermal tolerance experiments focus on biological mechanisms and physiological processes, and do not incorporate key ecological interactions, such as grazing and facilitation, which are known to mediate responses to ocean warming^64,99,100^. Despite these limitations, experimentally determined T_limit_ values have been show to be strongly correlated with empirical estimates derived from observations of mass mortalities in natural seagrass populations^26^, and case studies on species’ range contractions have demonstrated that T_limit_ values also correlate with observed population losses^9^. As such, experimentally derived T_limit_ values provide a useful tool for predicting population losses and range shifts of marine macrophytes.

The consolidation and exploration of experimentally-derived T_limit_ values represent a critical first step towards building more robust predictive tools for understanding the impacts of ocean extremes. Despite inherent complexities in real-world scenarios, experimentally derived T_limit_ values remain a powerful tool for predicting population losses and range shifts in the face of intensifying ocean warming and marine heatwaves. Moving forward, it is essential that the research community develops a standardised framework for assessing thermal limits. Ultimately, ocean temperatures are continuing to warm and extreme climatic events like marine heatwaves are becoming longer, more frequent and more severe^14,16^, increasing the thermal stress experienced by marine macrophytes in coastal ecosystems^17,37^. The ability to determine locations and populations where these critical foundation species are most at risk offers an opportunity for conserving these species and the ecological functions they underpin, and using ecologically relevant, experimentally-derived estimates of T_limit_ to inform mechanistic species distribution models will improve predictions of climate change impacts in the coming years and decades.

## Supplementary Information

Below is the link to the electronic supplementary material.

Supplementary Material 1

Supplementary Material 2

## Data Availability

All data are available in supplementary information.
