# Supplementary material for: Assessing the utility of experimentally derived upper thermal limits to predict vulnerability of marine macrophytes to future ocean warming
Source: Sci Rep. 2025 Nov 10;15:39320. doi: 10.1038/s41598-025-23074-z (PMC12603181; doi:10.1038/s41598-025-23074-z)
Supplement: Supplementary file 2 — Supplementary Material 2 [file 41598_2025_23074_MOESM2_ESM.docx]

Supplementary figure 1: Preferred Reporting Items for Systematic reviews and Meta-Analyses (PRISMA) flow diagram. Adapted from Page et al. 2021 and Lyford et al. 2025.


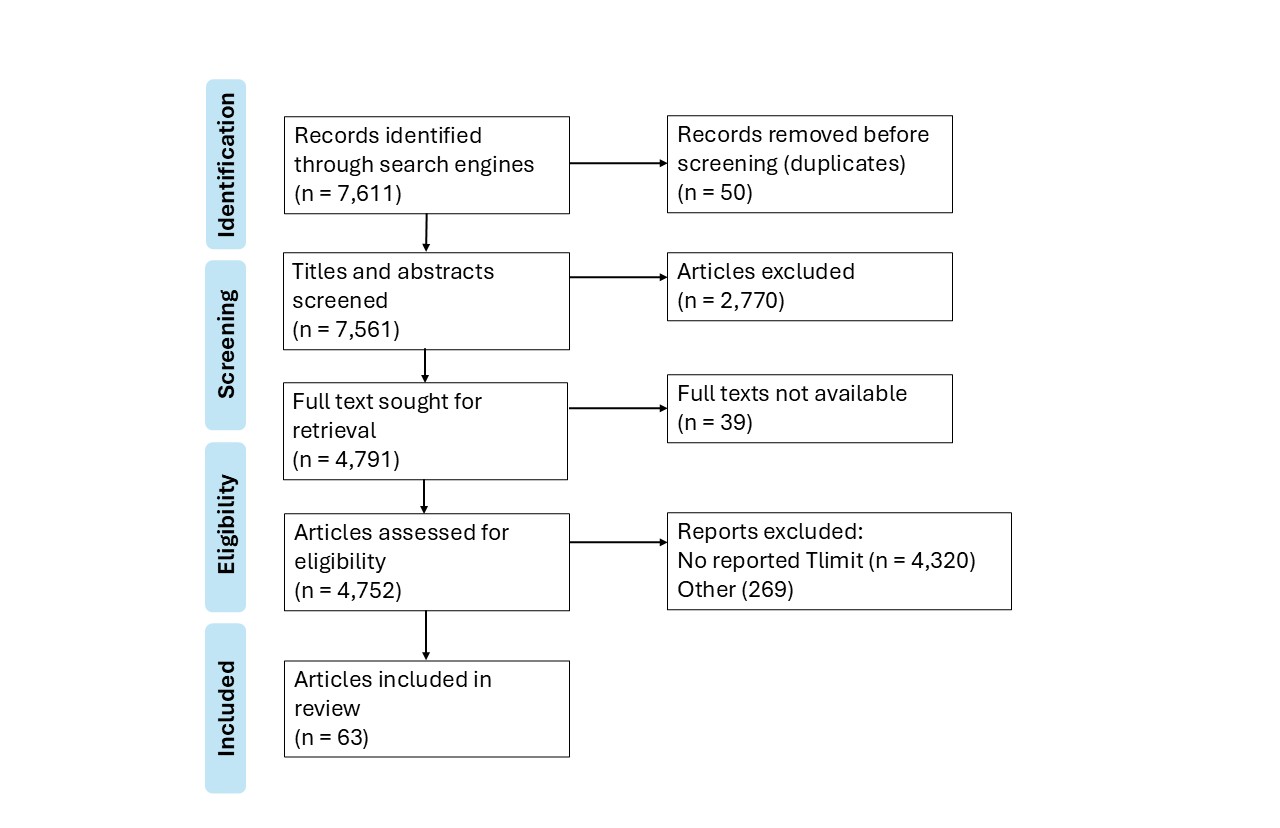


References:

Lyford, H. R., Romano, E. G., Sura, S. A., & Bittick, S. J. (2025). Understanding trends in Zostera research, stressors, and response variables: a global systematic review of the seagrass genus. *PeerJ*, *13*, e19209.

Page, M. J., McKenzie, J. E., Bossuyt, P. M., Boutron, I., Hoffmann, T. C., Mulrow, C. D., ... & Moher, D. (2021). The PRISMA 2020 statement: an updated guideline for reporting systematic reviews. *bmj*, *372*.
